# Analysis of the effects of group progressive resistance training on inflammatory markers, cardiovascular fitness parameters, and respiratory function in elderly patients with chronic obstructive pulmonary disease

**DOI:** 10.5937/jomb0-52323

**Published:** 2025-01-24

**Authors:** Li Chunyang, Sun Yijia

**Affiliations:** 1 Zhejiang Hospital, Intensive Care Unit, Hangzhou, China; 2 Zhejiang Hospital, Respiratory Department, Hangzhou, China

**Keywords:** chronic obstructive pulmonary disease, cardiopulmonary function, interleukin-8 (IL-8), Interleukin-18 (IL-18), interleukin-6 (IL-6), hronična opstruktivna bolest pluća, kardiopulmonalna funkcija, interleukin-8 (IL-8), interleukin-18 (IL-18), interleukin-6 (IL-6)

## Abstract

**Background:**

To investigate the effects of implementing group progressive resistance training on Maximal Oxygen consumption (VO2max), Maximum Ventilation per minute (VEmax), Maximal Oxygen pulse (O2pulsemax), Maximum Heart Rate (HRmax), and Modified Medical Research Council dyspnea scale (mMRC) in elderly patients with chronic obstructive pulmonary disease.

**Methods:**

A total number of 114 elderly patients with chronic obstructive pulmonary disease treated in the hospital from May 2022 to May 2024 were collected and divided into two groups based on different training methods. The conventional group (n=57) received routine rehabilitation training, while the organization group (n=57) received group progressive resistance training. Cardio - pulmonary Exercise Testing (CPET) parameters, serum inflammatory factors, lung function indicators, and mMRC score were compared between two groups before training, 2 weeks of training, and 4 weeks of training.

**Results:**

Before training, there was no significant difference between the two groups regarding training compliance, CPET parameters, inflammatory factors, and mMRC score. After 2-4 weeks of training, both groups showed improvements in training frequency, intensity, autonomous training, and increases in VO2MAX, VEmax, O2pulsemax, and HRmax. However, the organization group had higher scores in these areas and lower levels of inflammatory factors (IL-8, IL-18, IL-6, IL-12) and mMRC scores compared to the conventional group, with statistically significant differences (P<0.05).

**Conclusions:**

Group progressive resistance training can help improve the compliance of elderly patients with chronic obstructive pulmonary disease with training, reduce the body's inflammatory response, improve VO2MAX, VEmax, O2pulsemax, and HRmax levels, and alleviate breathing difficulties.

## Abstract (sr)

**Uvod:**

Cilj je bio da se istraže efekti primene grupnog progresivnog treninga otpora na maksimalnu potrošnju kiseonika (VO2max), maksimalnu ventilaciju po minuti (VEmax), maksimalni puls kiseonika (O2pulsemax), maksimalnu frekvenciju otkucaja srca (HRmax) i Modifikovanu skalu dispneje Medicinskog istraživačkog saveta (mMRC) kod starijih pacijenata sa hroničnom opstruktivnom bolešču pluča.

**Metode:**

Ukupno 114 starijih pacijenata sa hroničnom opstruktivnom bolešču pluča koji su lečeni u bolnici od maja 2022. do maja 2024. godine je podeljeno u dve grupe na osnovu različitih metoda treninga. Konvencionalna grupa (n = 57) je primala rutinski rehabilitacioni trening, dok je organizovana grupa (n = 57) imala grupni progresivni trening otpora. Parametri kardiopulmonalne testiranja (CPET), serumski inflamatorni faktori, indikatori funkcije pluča i mMRC skor upoređivani su između dve grupe pre treninga, posle 2 nedelje treninga i posle 4 nedelje treninga.

**Rezultati:**

Pre treninga nije bilo značajnih razlika između dve grupe u vezi sa usklađenošču treninga, parametrima CPET-a, inflamatornim faktorima i mMRC skorom. Nakon 2-4 nedelje treninga, obe grupe su pokazale poboljšanja u učestalosti treninga, intenzitetu, autonomnom treningu i povećanjima VO2max^ VEmax^ O2pulsemax ' HRmax. Međutim, organizovana grupa je imala više rezultate u ovim oblastima i niže nivoe inflamatornih faktora (IL-8, IL-18, IL-6, IL-12) i mMRC skorove u poređenju sa konvencionalnom grupom, sa statistički značajnim razlikama (P<0,05).

**Zaključak:**

Grupni progresivni trening otpora može pomoči u poboljšanju usklađenosti starijih pacijenata sa hroničnom opstruktivnom bolešču pluča sa treningom, smanjenju inflamatornog odgovora tela, poboljšanju nivoa VO2MAX, VEmax, O^pulsemax i HRmax, kao i ublažavanju poteškoća sa disanjem.

## Introduction

Chronic Obstructive Pulmonary Disease (COPD) is a common respiratory system disease in clinical practice, especially in the elderly population [1]. Patients often experience respiratory distress, decreased physiological function and exercise endurance after the onset of the disease, and decreased lung function and muscle mass are important factors leading to disease symptoms [2]. The prevalence of elderly diseases exceeds 30% [3]. However, in the context of the ageing population in China, the incidence rate of diseases can reach 26.3%, and the trend is gradually increasing [4]. The development of diseases affects patients’ physical health and reduces their ability to live. Rehabilitation training has been widely recognized as a vital part of promoting the rehabilitation of COPD patients, especially in strengthening their physical ability and Quality of Life (QOL) [5]. Applying exercise rehabilitation training to elderly COPD patients can promote the clinical symptoms and signs of the disease [6]. With the continuous increase in the elderly population in China, the rehabilitation of elderly COPD patients is highly valued in clinical practice. Within the scope of rehabilitation training, Group Progressive Resistance Training (GPRT) has attracted widespread clinical interest and research as a potential rehabilitation training method [7]. The implementation of GPRT emphasizes the combination of resistance training and aerobic training. The main purpose of training is to improve muscle strength and endurance, improve cardiopulmonary function, alleviate respiratory distress symptoms, and enhance the exercise capacity and QOL of elderly COPD patients [8]. However, despite numerous studies indicating the positive impact of this rehabilitation training method, further research and discussion are needed to determine its exact role and mechanism in the rehabilitation of elderly COPD patients. Therefore, exploring the impact of GPRT on respiratory function indicators in elderly patients with COPD is of great significance and is the subject of this study.

## Materials and methods

This experimental study was conducted on COPD patients referred to Zhejiang Hospital, Hangzhou, China, between May 2022 and May 2024 (ethical code: ZHH-2075CF5).

### Inclusion criteria

(A)Diagnosed as COPD based on imaging, laboratory tests, and clinical diagnostic evaluations, meeting the diagnostic criteria outlined in the 2013 Guidelines for the Diagnosis and Treatment of Chronic Obstructive Pulmonary Disease [9]; (B)The condition is in a stable phase, with the researchers used forced expiratory volume in 1 second /Forced vital capacity (FEV_1_/FVC)<70%; (C) Capable of regularly cooperating with relevant inspections; (D) Normal audio-visual, reading and writing, communication, and cognitive functions.

### Exclusion criteria

(A) Obstructive pulmonary ventilation dysfunction caused by other diseases; (B) Concomitant pulmonary diseases such as active tuberculosis, bronchiectasis, and lung cancer; (C) Combined liver and kidney dysfunction; (D) Concomitant severe heart disease and arrhythmia; (E) Merge blood system diseases.

### Research method

CG: Implement Routine Rehabilitation Training (RRT). Rehabilitation trainers assist in developing training plans, and responsible nurses are responsible for explaining and supervising the implementation of training. The specific operation method is as follows: (A) Lip contraction breathing training: Take a sitting or standing position to relax the body; Guide the patient to slightly contract their lips, close them, leave small gaps, and slowly inhale deeply through their nose, maintaining breathing for 1–2s; Then the lips appear in a whistling shape, and when exhaling, resistance is applied at the lip position to achieve an inhalation to exhalation time ratio of 1:2. (B) Abdo minal breathing training: Take a lying position and relax the body; Place the hands in the abdominal position to feel the ups and downs of the abdomen. Firstly, slowly inhale through the nose while slowly bulging the abdomen for 5–10s. Exhale while the abdomen is sunken; try to exhale all the gas as much as possible. Both methods are repeated 10–20 times to form a group, with 2 groups trained daily, 5 times a week, and continuous training for 4 weeks.

OG: Implement GPRT based on RRT. (A) Establish a training group: Divide 5–10 patients with small differences in their condition and individual differences into a training group through evaluation and investigation. Before implementing the training, the rehabilitation trainer and responsible nurse jointly use multimedia videos to guide their training methods, frequency, coordination, and precautions. Guide patients in raising questions about training during guidance and then provide answers to ensure that each group member can master the implementation methods of training. (B) Progressive resistance breathing training: Implement training using lung function training equipment. Before training, use the pressure valve head of the instrument to adjust respiratory resistance. Then, after adjusting their breathing, the patient’s teeth should be filtered, their lips should be closed, and they should undergo inspiratory muscle training. They should try to inhale slowly to the maximum extent possible. After a 3s interval, slowly exhale until fully exhaled, and perform expiratory muscle training similarly. The same resistance training is repeated 10–20 times. Through observation and evaluation, when the patient’s training score reaches 80 points as the standard, the resistance of the training instrument can be increased by one level (based on 3 cm H_2_O as the standard), achieving the goal of progressive training. Breathing and exhaling muscle training alternate, with 30 sessions per group for 10 minutes each time. Train 5 times a week for 4 weeks. (C) Progressive resistance exercise training: The main training methods include knee extension, knee flexion, sitting chest expansion, sitting up, and sitting forward push. Train each action 6–8 times as a group, with a duration of 3 s each time. By evaluating the patient’s condition, guide them to gradually increase the frequency and duration of training to achieve maximum patient tolerability. Train 2 groups daily for 4 weeks.

### Observation indicators and evaluation

Training Compliance: The Rehabilitation Training Compliance Scale for Patients After Pacemaker Implantation [10] was used as a reference, and the department conducted a self-made scale for investigation. The scale consists of 4 aspects and 20 questions. It adopts a 1–5 level scoring method, totalling 0–100 points. The higher the score, the greater the patient’s compliance with the training.

Cardiopulmonary Exercise Testing (CPET): The attending physician and responsible nurse assess jointly. Before the test, the patient was instructed not to engage in other activities for 24 hours and not to drink or eat for the first hour. The test was conducted using a powered bicycle. After calibration, the symptom self-limiting maximum exercise load increment test is applied in accordance with the patient’s status. The mask, electrocardiogram lead, and blood pressure cuff are connected, and a warm-up is performed before the test. Then, a power increment training test was conducted on a power bicycle, with a resting time of 1 minute at 0W power and a warm-up time of 2 minutes at 0W power. The starting power of the treadmill load was 5 W. The exercise load increases in power according to symptom limits of 5–20 W/min, maintaining a speed of 58–62 r/min until the maximum exercise load occurs. Then, recover at 0W power until the indicators related to oxygen uptake, heart rate, and carbon dioxide excretion return to the platform level, and the experiment can be terminated. During exercise, VO_2max_, VE_max_, O_2_pulse_max_, and HR_max_ were evaluated, and all patients achieved maximum exercise load.

*Inflammatory factor indicators*: Serum samples are obtained by the responsible nurse, and inflammatory indicators such as Interleukin-8 (IL-8), Inter leukin-18 (IL-18), Interleukin-6 (IL-6), and Inter leukin-12 (IL-12) were tested in the laboratory. Instruct the patient to take 5 mL of venous blood on an empty stomach, let it stand for 30 minutes, and then place it in a frozen centrifuge at 4°C. Centrifuge at a radius of 6cm and a speed of 3000 r/min for 10 min to gain the supernatant. Enzyme-linked immuno sorbent assay (ELISA) and corresponding test kits were used to determine inflammatory markers.

*Respiratory difficulties*: With the assistance of nursing staff, the patient evaluates their breathing difficulties using the Modified Medical Research Council (mMRC) [11]. The scale has four levels in total. Level 0: Difficulty breathing during intense activity. Level 1: Difficulty breathing while walking briskly on flat ground or climbing gentle slopes. Level 2: Due to difficulty breathing, walking slower than peers on flat ground requires stopping to rest. Level 3: Walking on flat ground for a distance of 100 meters or walking for a few minutes requires stopping to rest and catch your breath. Level 4: Unable to walk away from home, experiencing difficulty breathing while dressing or undressing.

### Statistical data analysis

All data were entered into the statistical software SPSS 25.0, and the measurement data that followed a normal distribution were described as mean ± standard deviation (x̄±s). An independent sample t-test was performed between groups, and the sample t-test was paired with the groups. Counting data was described using examples (n) and rates (%), and the chi square χ^2^ test was conducted between groups. *P*<0.05 indicates statistical significance in data comparison.

## Results

**Figure 1:**
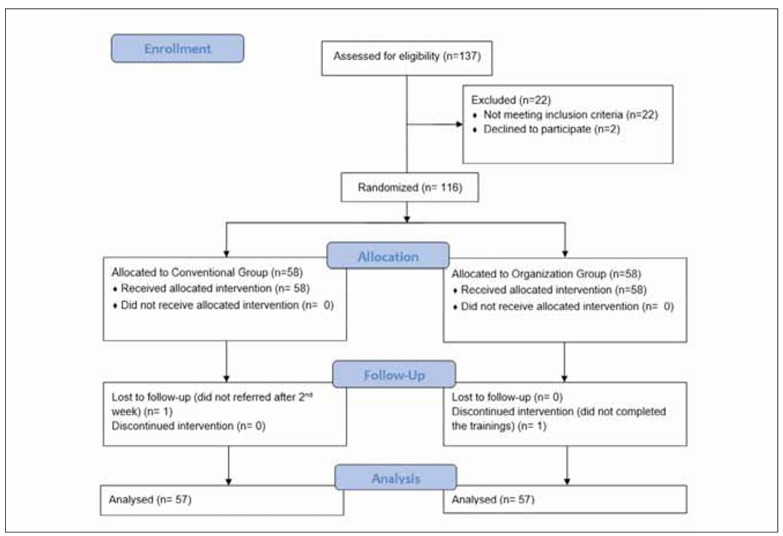
CONSORT flowchart of study.

Figure 1 shows the flow diagram of the study population participating. A total of 137 participants were assessed for eligibility; 22 were excluded due to not meeting inclusion criteria, and 2 declined to participate. The remaining 113 participants were randomized, with 58 allocated to the Organization Group and 58 to the Conventional Group. All participants in both groups received their allocated intervention, with no instances of non-receipt. One participant in the Conventional Group was lost to follow-up after the 2nd week, while one participant in the Organization Group discontinued the intervention due to not completing the training. No participants in either group were lost to follow-up or discontinued the intervention for other reasons.

Information on 114 elderly COPD patients treated at the hospital from May 2022 to May 2024 was collected and segmented into Conventional Group (CG) and Organization Group (OG) based on different training methods, with 57 patients in each group.CG: 26 males and 31 females, aged from 60 to 68, with an average of (64.37±2.318); The course of the disease is 1–5 years, with the mean coarse of (3.1±1.06); Body Mass Index (BMI): 22–26 kg/m^2^, with an average BMI of (24.15±1.03) kg/m^2^; Complications: 16 cases of hypertension, 13 cases of diabetes, 15 cases of hyperlipidemia, 13 cases of Coronary Heart Disease (CHD); 38 cases had a history of smoking and 19 cases had a history of drinking alcohol. OG: 26 males and 31 females, aged 60–68 years, with an average age of (64.3 ±2.318) years; The disease course is 1–5 years, with an average course of (3.15±1.06) years; BMI: 22–26 kg/m^2^, with an average BMI of (24.15±1.03) kg/m^2^; Complications: 18 cases of hypertension, 15 cases of diabetes, 16 cases of hyperlipidemia, 8 cases of CHD; 35 cases of smoking history and 22 cases of drinking history. There was *P*>0.05 between the two groups of data.

Comparative data revealed significant improvements in various parameters following 2 and 4 weeks of training. Specifically, training frequency, intensity, and autogenic training showed significant increases in both groups at 2 and 4 weeks (p<0.01), with the OG exhibiting greater improvements. Self-training reports also improved significantly in both groups at 2 and 4 weeks (p<0.01). Inflammatory markers IL-8, IL-18, IL-6, and IL-12 decreased significantly in both groups at 2 and 4 weeks (p<0.05), with the OG showing greater reductions. Cardiovascular parameters, including VO_2max_, VE_max_, O_2pulsemax_, and HR_max_, increased significantly in both groups at 2 and 4 weeks (p<0.05), with the OG exhibiting greater improvements. The modified Medical Research Council (mMRC) score, a measure of dyspnea, decreased significantly in both groups at 2 and 4 weeks (p<0.01), with the OG showing a greater reduction (Table 1).

**Table 1:** Comparative data in two groups in pre-training, at 2 and 4 weeks of training (x̄±s). Note: * represents a comparison with pre-training in this group, P<0.05; # represents a comparison with training for week 2 in this group, P<0.05.

	Group	CG	OG	t	P
	n	57	57		
Training frequency	Pre-training	10.89±1.35	10.81±1.28	0.325	0.746
	After 2 weeks	12.19±1.41*	14.94±1.65*	9.566	<0.01
	After 4 weeks	17.86±3.12*#	21.42±3.81*#	5.458	<0.01
Training intensity	Pre-training	9.13±1.08	9.29±1.11	0.78	0.437
	After 2 weeks	11.89±1.32*	15.46±1.51*	13.438	<0.01
	After 4 weeks	18.38±2.57*#	20.00±2.60*#	3.346	0.001
Autogenic training	Pre-training	8.66±1.54	8.81±1.36	0.551	0.583
	After 2 weeks	11.58±2.22	13.47±2.69	4.091	<0.01
	After 4 weeks	16.74±3.16	18.37±3.71	2.525	0.013
Self-training report	Pre-training	7.39±1.16	7.15±1.28	1.049	0.297
	After 2 weeks	9.38±2.49	12.47±2.81	6.114	<0.01
	After 4 weeks	14.67±3.10	17.27±3.61	4.125	<0.01
IL-8 (mg/L)	Pre-training	1004.37±201.85	1004.41±201.79	0.001	0.999
	After 2 weeks	923.60±181.35*	847.23±175.26*	2.285	0.024
	After 4 weeks	783.11±179.22*#	491.86±161.05*#	9.126	<0.01
IL-18 (mg/L)	Pre-training	125.67±21.09	125.71±21.30	0.01	0.992
	After 2 weeks	117.34±20.61*	106.80±19.54*	2.802	0.006
	After 4 weeks	112.77±18.64*#	102.34±17.55*#	2.987	0.004
IL-6 (mg/L)	Pre-training	303.24±40.29	303.67±41.91	0.056	0.956
	After 2 weeks	231.06±38.64*	215.18±34.69*	2.309	0.023
	After 4 weeks	176.34±25.43*#	127.61±23.08*#	10.713	<0.01
IL-12 (pg/L)	Pre-training	130.49±22.34	130.71±21.98	0.053	0.958
	After 2 weeks	117.34±21.09*	108.29±19.67*	2.369	0.02
	After 4 weeks	107.24±20.07*#	88.37±15.94*#	5.559	<0.01
VO_2max_ (mL/min)	Pre-training	0.81±0.15	0.83±0.14	0.736	0.463
	After 2 weeks	1.32±0.18*	1.64±0.20*	8.979	<0.01
	After 4 weeks	1.58±0.21*#	1.76±0.23*#	4.363	<0.01
VE_max_ (L/min)	Pre-training	50.22±10.37	50.18±10.76	0.02	0.984
	After 2 weeks	64.37±11.69*	69.86±12.07*	2.467	0.015
	After 4 weeks	68.29±13.26*#	74.44±13.27*#	2.475	0.015
O_2_pulse_max_ (mL/beat)	Pre-training	7.69±1.26	7.95±1.14	1.155	0.25
	After 2 weeks	8.99±1.91*	10.29±2.01*	3.54	0.001
	After 4 weeks	9.97±2.00*#	11.58±2.47*#	3.825	<0.01
HR_max_ (times/min)	Pre-training	157.66±15.37	157.81±15.29	0.052	0.958
	After 2 weeks	161.37±16.57*	168.34±16.91*	2.223	0.028
	After 4 weeks	165.27±17.31*#	172.09±17.55*#	2.089	0.039
mMRC score	Pre-training	3.05±0.75	3.10±0.69	0.37	0.712
	After 2 weeks	2.54±0.63*	2.02±0.53*	4.769	<0.01
	After 4 weeks	1.86±0.41*#	1.20±0.29*#	9.922	<0.01

## Discussion

By describing the introduction, it is clear that the initial aim of implementing this paper is to explore the impact of GPRT on elderly COPD patients, with a particular focus on indicators such as VO2max, VEmax, O2pulsemax, HRmax, and mMRC. This study proposes the physiological and functional challenges faced by elderly COPD patients in disease treatment, as well as the hypothesis that implementing GPRT may positively impact their training compliance, symptom improvement, and overall health. The background of this study highlights the importance of COPD as a chronic respiratory disease, which is more common in the elderly population and often accompanied by adverse problems such as difficulty breathing and decreased muscle physical and aerobic capacity. These issues may lead to a decrease in the patient’s ability to live [12]. O’Shea et al. [13] emphasized the potential of resistance training in improving respiratory efficiency, muscle strength, and cardiopulmonary function in multiple aspects. However, the application effect on elderly COPD patients is still unclear. The main goal is to verify the effectiveness of the GPRT application in elderly COPD patients. This study particularly focuses on key physiological indicators, including VO_2max_, VE_max_, O_2pulsemax_, HR_max_, IL-8, IL-18, IL-6, IL-12, and mMRC indicators. By evaluating the above indicators, it is attempted to understand whether GPRT can improve cardiovascular function, inflammatory levels, and respiratory status in elderly COPD patients [14].

The conclusion drawn from this study is that GPRT can help improve the compliance of elderly COPD patients with rehabilitation training, leading to an increase in their reported scores for training frequency, training intensity, autonomous training, and self-training. This indicates that the compliance of elderly COPD patients with rehabilitation training has significantly improved. GPRT can promote patient interaction, share training experiences, strengthen their confidence, and enhance their interest in training. In a group environment, patients may feel more motivated, which motivates them to participate more actively in training [15]. Group training has a certain structure and time, making it easier for patients to establish regularity and standardization in training and helping them develop healthy habits [16]. The results of this study are consistent with those of Kongsgaard et al. [17], which indicated that elderly COPD patients are more likely to maintain exercise compliance in a group environment. This viewpoint suggests that GPRT can provide an interactive, supportive, and supervised environment for elderly COPD patients, which can help improve their compliance. The difference between the research results of Mehani et al. [18] lies in the fact that the training compliance of elderly COPD patients is influenced by individual differences, physical health status, psychological and emotional factors, as well as individual exercise preferences, and not solely depends on the training environment. Therefore, some patients may benefit from implementing GPRT, while some patients may not have significant training effects and may be more suitable for other forms of rehabilitation plans. This indicates that personalization and comprehensive practical situations are crucial to developing effective rehabilitation training plans for elderly COPD patients [19].

Regular rehabilitation exercise training, such as resistance, aerobic exercise, and respiratory training, can improve cardiovascular function and exercise endurance in elderly COPD patients, help alleviate breathing difficulties, and improve QOL [20]. Applying various exercise rehabilitation plans can produce good benefits for elderly COPD patients [20]. Aerobic exercise, muscle strength training, and respiratory training can help improve exercise endurance, lung function, and QOL for elderly COPD patients [12].

## Conclusion

In summary, implementing GPRT in elderly COPD patients significantly improves their compliance with training, promotes overall body recovery, reduces inflammatory reactions, promotes the recovery of cardiovascular function indicators, and improves respiratory distress symptoms. The application of GPRT in elderly COPD patients is a promising research direction that can focus on the long-term effects of training implementation and help patients determine the impact of training on long-term health and QOL. The study can further focus on the positive impact of GPRT on the QOL of elderly COPD patients by exploring a GPRT plan tailored to the individual situation and condition of each COPD patient, thereby providing medical professionals with more information and guidance on better management of elderly COPD patients. Although this study has achieved certain results, there are still certain limitations. For example, selecting a small sample size may lead to variability in the research results and increase the risk of errors in multiple statistical test types. This study did not select long-term QOL indicators for evaluation, which resulted in the inability to capture the long-term rehabilitation effects of patients and comprehensively evaluate the training value. Therefore, in subsequent research, increasing the sample size and long-term evaluation indicators is necessary to understand GPRT’s effectiveness better and provide an important reference for elderly COPD patients to implement rehabilitation training.

## Dodatak

### Conflict of interest statement

All the authors declare that they have no conflict of interest in this work.

## References

[1] Soriano J B, Marín J M, Celli B R (2023). Post-bronchodilator spirometry in chronic obstructive pulmonary disease. Lancet Resp Med.

[2] Fahy J V, Locksley R M (2023). Immune treatment tackles chronic obstructive pulmonary disease. Nat.

[3] Diaz A A, Orejas J L, Grumley S, Nath H P, Wang W, Dolliver W R, et al (2023). Airway-occluding mucus plugs and mortality in patients with chronic obstructive pulmonary disease. Jama.

[4] Motahari A, Barr R G, Han M K, Anderson W H, Barjaktarevic I, Bleecker E R, et al (2023). Repeatability of pulmonary quantitative computed tomography measurements in chronic obstructive pulmonary disease. Am J Respir Crit Care Med.

[5] Feldman W B, Avorn J, Kesselheim A S, Gagne J J (2023). Chronic obstructive pulmonary disease exacerbations and pneumonia hospitalizations among new users of combination maintenance inhalers. JAMA Intern Med.

[6] Lacasse Y, Casaburi R, Sliwinski P, Chaouat A, Fletcher E, Haidl P, et al (2022). Home oxygen for moderate hypoxaemia in chronic obstructive pulmonary disease: A systematic review and meta-analysis. Lancet Resp Med.

[7] Rustam S, Hu Y, Mahjour S B, Rendeiro A F, Ravichandran H, Urso A, et al (2023). A unique cellular organization of human distal airways and its disarray in chronic obstructive pulmonary disease. Am J Respir Crit Care Med.

[8] Valenzuela T (2012). Efficacy of progressive resistance training interventions in older adults in nursing homes: A systematic review. Journal of the American Medical Directors Association.

[9] Koblizek V, Chlumsky J, Zindr V, Neumannova K, Zatloukal J, Zak J, et al (2013). Chronic obstructive pulmonary disease: Official diagnosis and treatment guidelines of the Czech Pneumological and Phthisiological Society: A novel phenotypic approach to COPD with patient-oriented care. Biomed Paper.

[10] Madapoosi S S, Cruickshank-Quinn C, Opron K, Erb-Downward J R, Begley L A, et al (2022). Lung microbiota and metabolites collectively associate with clinical outcomes in milder stage chronic obstructive pulmonary disease. Am J Respir Crit Care Med.

[11] Qian F, van den Boom W, See K C (2023). Real-world evidence challenges controlled hypoxemia guidelines for critically ill patients with chronic obstructive pulmonary disease. Intens Care Med.

[12] Cabrera López C, Sánchez Santos A, Lemes Castellano A, Cazorla Rivero S, Breña Atienza J, González Dávila E, et al (2023). Eosinophil subtypes in adults with asthma and adults with chronic obstructive pulmonary disease. Am J Respir Crit Care Med.

[13] O'Shea S D, Taylor N F, Paratz J D (2007). Qualitative outcomes of progressive resistance exercise for people with COPD. Chronic Respiratory Disease.

[14] Abd El-Kader S M, Al-Jiffri O H, Al-Shreef F M (2016). Plasma inflammatory biomarkers response to aerobic versus resisted exercise training for chronic obstructive pulmonary disease patients. African Health Sciences.

[15] Hogg L, Grant A, Garrod R, Fiddler H (2012). People with COPD perceive ongoing, structured and socially supportive exercise opportunities to be important for maintaining an active lifestyle following pulmonary rehabilitation: A qualitative study. J Physiotherap.

[16] Rojas J C, Chokkara S, Zhu M, Lindenauer P K, Press V G (2023). Care quality for patients with chronic obstructive pulmonary disease in the readmission penalty era. Am J Respir Crit Care Med.

[17] Kongsgaard M, Backer V, Jørgensen K, Kjær M, Beyer N (2004). Heavy resistance training increases muscle size, strength and physical function in elderly male COPD-patients-a pilot study. Respiratory Medicine.

[18] Mehani S H (2017). Comparative study of two different respiratory training protocols in elderly patients with chronic obstructive pulmonary disease. Clin Interv Aging.

[19] Panton L B, Golden J, Broeder C E, Browder K D, Cestaro-Seifer D J, Seifer F D (2004). The effects of resistance training on functional outcomes in patients with chronic obstructive pulmonary disease. Erup J Applied Physiol.

[20] Zambom-Ferraresi F, Cebollero P, Gorostiaga E M, Hernández M, Hueto J, Cascante J, Rezusta L, Val L, et al (2015). Effects of combined resistance and endurance training versus resistance training alone on strength, exercise capacity, and quality of life in patients with COPD. J Cardiopulm Rehabil Prev.

